# Pre-operative expectations in patients with endometriosis – a qualitative interview study

**DOI:** 10.1186/s12905-025-03686-3

**Published:** 2025-04-28

**Authors:** Nina Hirsing, Yvonne Nestoriuc, Olaf Buchweitz, Ann-Katrin Meyrose

**Affiliations:** 1 Clinical Psychology and Psychotherapy, Helmut-Schmidt-University, University of the Federal Armed Forces Hamburg, Hamburg, Germany; 2 Institute of Systems Neuroscience, University Medical Centre, Hamburg-Eppendorf, Germany; 3 Frauenklinik an der Elbe, Centre of Surgical Endoscopy and Endometriosis, Hamburg, Germany; 4 Department of Child and Adolescent Psychiatry, Psychotherapy, and Psychosomatics, University Medical Centre Hamburg-Eppendorf, Hamburg, Germany

**Keywords:** Placebo, Nocebo, Treatment expectation, Laparoscopy, Barriers

## Abstract

**Background:**

Expectations determine treatment outcomes in several medical conditions. The significance of expectations for treatment outcomes in patients with endometriosis remains unknown. Endometriosis is a painful and debilitating disease that negatively affects quality of life. Up to 30% of surgically treated patients report persistent post-operative complaints and pain disability without sufficient medical explanation, indicating the impact of non-medical factors on treatment outcomes.

**Aim:**

The present qualitative study aimed to describe and understand pre-operative patient expectations, facilitators of and barriers to positive treatment outcomes.

**Method:**

As part of a large mixed-method cohort study, a subsample of *N* = 33 patients with endometriosis were interviewed before laparoscopy. Structured content analysis was performed.

**Results:**

Positive expectations included significant improvement or absence of complaints, receiving a diagnosis, and subsequently improved health-related quality of life. However, patients also reported negative expectations such as invalidation of their experience, persistence of complaints, or post-operative side effects. Patients perceived positive expectations as facilitators for positive treatment outcomes. Further facilitators included enhanced patient and treatment information, gynaecologists specialized in endometriosis, and greater awareness of endometriosis. Perceived barriers to good post-operative quality of life included post-operative pain and scarring, insufficient rest, avoidance behaviour, and stress.

**Conclusion:**

Positive and negative expectations coexisted. Positive expectations suggest that participants place much hope in laparoscopy. However, these positive expectations may exceed probable treatment outcomes for some patients. Negative expectations were also expressed and constituted a risk for nocebo effects. Further identified facilitators and barriers show that patients are very clear about what is helpful or not for their health-related quality of life after laparoscopy. Patient and treatment information may be enhanced to prevent unrealistic treatment expectations and nocebo effects.

**Supplementary Information:**

The online version contains supplementary material available at 10.1186/s12905-025-03686-3.

The trial is preregistered at ClinicalTrials.gov (ID NCT05019612), registration date August 25th 2021.

## Background

Over recent decades, increasing evidence suggests that patient expectations substantially determine treatment outcomes across various medical conditions [1]. As a result, expectations have been addressed in treatment processes, leading to optimized treatment outcomes, i.e. in heart surgery or breast cancer patients [2, 3].

Endometriosis is a common and debilitating gynaecological disease that affects 4.4% of the female population [4]. It is defined as inflammatory lesions of endometrial-like tissue outside the uterine cavity [5]. The most frequently reported symptoms of endometriosis include dysmenorrhoea, pelvic pain, dyschezia, dysuria, dyspareunia, and infertility [6]. Symptoms’ clinical presentation, frequency, and intensity vary, resulting in delayed diagnosis [7]. Endometriosis has a significant negative impact on patients, affecting their social and professional lives, overall quality of life, and relationships [8–10]. Beyond that, people diagnosed with endometriosis demonstrate higher rates of anxiety, depression, and general emotional distress compared to the female general population [11, 12], underscoring the substantial psychological burden imposed by the disease.

If conservative hormonal treatments did not yield sufficient symptom relief, international treatment guidelines recommend laparoscopic surgery for endometriosis-related chronic pain and infertility [13, 14]. Nevertheless, 20 to 30% of treated patients experience significant post-operative symptom persistence and disability [15–17]. Deep infiltrating endometriosis [18] and concomitant adenomyosis [19, 20] are considered medical risk factors for ongoing post-operative complaints. Additionally, biopsychological factors, such as depression, anxiety, pain catastrophizing, and younger age, have previously been identified as significant predictors of persistent symptoms in patients with endometriosis [17, 21, 22]. However, these factors do not explain symptom persistence entirely, suggesting that additional factors may be crucial for treatment outcomes in women with endometriosis.

As expectations are relevant for treatment outcomes in several medical conditions, they may also serve as influencing factors in people with endometriosis. Initial evidence suggests that expectations may be relevant for this patient group: Placebo-response rates of up to 32% have been documented in patients with endometriosis following sham laparoscopy [23], indicating that their perception of receiving treatment may have contributed to the outcome. Furthermore, patients who had just undergone laparoscopy for biopsy extraction showed pain improvement comparable to patients who received both biopsy and endometriosis extraction in a single procedure [16, 24]. It is thus important to explore and understand the expectations of patients with endometriosis more precisely. Unsatisfactory treatment outcomes after laparoscopy may be affected by nocebo effects resulting from negative expectations [25, 26].

Qualitative research has identified factors such as social support and pain management as facilitators for positive treatment outcomes after surgery. Mental health issues and lack of social and professional support have been identified as barriers to positive outcomes following surgery [27]. However, there is a lack of research that specifically examines the facilitators of and barriers to post-operative quality of life in patients with endometriosis. To the best of our knowledge, this study is the first to investigate expectations, facilitators, and barriers to quality of life in women with endometriosis following laparoscopy. Semi-structured interviews were conducted with patients with endometriosis to address the following research questions:

1. Which endometriosis-related complaints and disabilities do patients name?
2. Which positive and negative expectations do patients name regarding laparoscopy and post-operative quality of life?
3. Which facilitators and barriers do patients perceive regarding post-operative quality of life?

## Methods

### Study design

This interview study was conducted within a mixed-method longitudinal clinical cohort study with one pre- and eight post-operative assessments and an ambulatory assessment. (ClinicalTrials.gov identifier: NCT05019612, study protocol: 10.1136/bmjopen-2022-067497). Study’s conception, conduct, and reporting followed consolidated criteria for qualitative research (COREQ, [28]). Interviews were taken pre- and post-operatively. This study refers to the pre-operative interviews only.

### Recruitment and sample

We conducted semi-structured interviews with 35 patients with a clinical indication for laparoscopy because of suspected endometriosis. Patients scheduled for laparoscopy at a specialist centre for surgical endoscopy and endometriosis in Germany (Frauenklinik an der Elbe) were informed about the clinical cohort study by telephone. Interview recruitment was included at the end of the clinical cohort study’s baseline assessment between 21 August 2021 and 31 May 2022. Interested patients provided their mobile phone numbers to be contacted by the study team for detailed study information and informed consent.

#### Inclusion and exclusion criteria

Patients had to fulfil the following criteria to be included in the interview study: (1) aged 18 years or older, (2) endometriosis-related complaints with or without an unmet wish to have children, (3) proficiency in written and spoken German, (4) female sex, (5) informed consent for study participation, (6) indication for laparoscopy, (7) endometriosis visually diagnosed by clinicians, two further criteria confirmed post-operatively: (8) complete excision of endometrial tissue, and (9) benign biopsy result of endometrial tissue.

### Data collection

Interviews were conducted via telephone, using a semi-structured interview guide with 14 open-ended questions (see supplementary Material 2, Document 2). Interviews were devised by NH (M.Sc. psychologist, PhD student in Clinical Psychology), AKM (PhD, M.Sc. psychologist), and YN (Professor of Clinical Psychology and Psychotherapy, licensed psychotherapist). Interviews were conducted at the authors’ workplace by NH, AKM or trained psychology students one week before the laparoscopy. All Interviews were audio recorded. Informed consent was obtained verbally and in writing. The mean interview time was 16:33 min, ranging from 7:02 to 37:29 min. At the end of the interviews, sociodemographic and prior treatment experiences were explored using a ten-item questionnaire lasting approximately 5 min. Inclusion criteria seven to nine were checked after laparoscopy, leading to some patients and their interviews being excluded from the analysis subsequently.

### Data analysis

We conducted a structural content analysis using the qualitative research software MaxQDA 2023. NH and AKM independently coded the transcripts using the following six concept-driven top-level thematic codes: *complaints and disability (1 & 2)*, *positive and negative expectations (3 & 4)*, as well as *facilitators and barriers (5 & 6).* These codes were directly related to the research questions and assigned in multiple iterations. Subsequently, data-driven sub-level thematic codes were developed in accordance with the principle of data saturation and assigned through multiple cycles. Any divergent code was discussed among NH, AKM and other colleagues, and a complete consensus was reached.

Code definitions and preliminary findings were refined during multiple peer debriefing sessions with colleagues and psychology students to enhance the study’s validity. NH, AKM, and a dedicated minute taker conducted a participant checking session. This took place as a recorded online group discussion where the research findings were presented to five study participants, who were given the opportunity to discuss our findings from their point of view, improve clarity, and suggest missing aspects. Final findings and quotations were translated into English with the aid of a colleague who is a native speaker of English as well as a professional translation service (see supplementary Material 1, Table 1).

## Results

### Sociodemographic and clinical data

A total of *N* = 35 patients with suspected endometriosis participated in the qualitative study. Two participants were excluded post-operatively because their endometriosis diagnosis was not confirmed (see supplementary Material 3, Fig. 3). Ultimately, *N* = 33 participants were included for content analysis (Table 1). Among them, 33 (97.0%) identified as female and one as non-binary (3.0%). Age ranged from 21 to 43 years (*M* = 30.48, *SD* = 5.81). Most participants had a university degree (42.4%) or a general qualification for university entrance, i.e., a German Abitur (27.3%). The majority of participants were diagnosed with minimal (43.3%) or mild (26.7%) endometriosis in accordance with the rASRM score criteria (revised American Society of Reproductive Medicine score [29]), while the remaining participants were diagnosed with moderate (16.7%) or severe (3.3%) endometriosis. Duration of symptoms ranged from 1.5 to 30 years (*M* = 9.78, *SD* = 8.35). Of the 33 participants, seven (27.3%) reported having previous treatment experiences with laparoscopy within the last 12 months, and 17 participants (51.5%) reported previous experience with other endometriosis-related treatments (e.g., hormone therapy).

**Table 1:** Sociodemographic and clinical characteristics of *N* = 33 participants with endometriosis

	n (%)	M	SD	Range
**Gender**				
Female	32 (97.0)			
Non-binary	1 (3.0)			
**Age** in years		30.48	5.81	21–43
**Nationality**				
German	33 (100.0)			
Immigrant background*	6/24 (18.2)			
**Level of education**				
Lower secondary school	1 (3.0)			
Secondary school	8 (24.2)			
Qualification for higher education	9 (27.3)			
University degree	14 (42.4)			
Not specified	1 (3)			
**Reason for laparoscopy**				
Endometriosis-related complaints	30 (90.9)			
Endometriosis-related complaints with an unmet wish to have children/Infertility	3 (9.1)			
**Treatment experiences with laparoscopy within the last 12 months (yes)**	7 (27.3)			
**Other endometriosis-related treatment experiences (yes)**	17 (51.5)			
**Stage of endometriosis (rASRM)**				
Minimal	16 (47.1)			
Mild	9 (26.5)			
Moderate	5 (14.7)			
Severe	1 (3.3)			
Not specified	2 (5,9)			
**Duration of symptoms (months)**	29 (89.9)	9.78	8.35	1.5–30

Note. *M* = mean; *SD* = standard deviation; rASRM = revised American Society for Reproductive Medicine Score; immigrant background, i.e., participants or one or both parents were not born in Germany, *n = 24, item was not included until some interviews had already been conducted

### Endometriosis-related complaints and disability

Patients reported a wide range of endometriosis-related complaints and disability, summarized in Table 2. Cardinal complaints are highlighted in bold font.

**Table 2:** Reported endometriosis-related complaints and disability

Complaint	Areas affected by disability
Cyclic vaginal thrush	Ability to work
Dizziness	Feeling of agency over one’s body and body image
**Dysmenorrhea**	Mental health
**Dysuria & Dyschezia**	Mobility
**Dyspareunia**	Nutrition
Gastrointestinal symptoms	Physical fitness
Headache	Reliance on the sympathy and understanding of one’s social environment
**Unmet wish to have children/Infertility**	One’s social environment
Metrorrhagia	Romantic relationships
Nausea and vomiting	Self-esteem
Ovulation pain	Sexual intimacy
**Pelvic pain**	Sleep
Raised temperature/fever	Social, family, and leisure activities
Shoulder, leg, and back pain	Nutrition
Sweating	
Vertigo	
Water retention	

Note. Sorted alphabetically. Cardinal complaints are highlighted in bold font

**Fig. 1:**
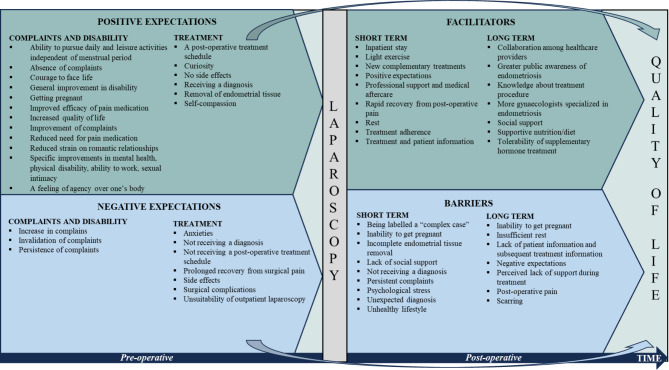
Overview of pre-operative expectations regarding laparoscopy, facilitators for and barriers to post-operative quality of life

Qualitative findings are sorted thematically and documented with sub-level codes in bold font. Quotations are shown in italics. Figure 1 presents a schematic overview of our findings.

## Expectations

Positive and negative expectations are sorted thematically by the categories of *complaints and disability*, and *treatment*.

### Positive expectations

#### Complaints and disability

Most participants expected **complaints improvement** following laparoscopy, but some expected an absolute **absence of complaints**.

‘But I just hope that I will be in less pain’ (P28; f, 27y).

‘I really have very high expectations that I will not be in pain anymore’ (P2, f, 33y).

Another positive expectation was **to get pregnant** and experience motherhood.

‘Maybe finally getting pregnant […] and finally being able to be a mother’ (P27, f, 26y).

While some participants expected **general improvements in disability**, others cited specific improvements in areas such as **mental health**, **physical disability**, **ability to work**, and **sexual intimacy**. Additionally, participants expected **reduced strain on their romantic relationships**.

‘So, yes, a positive outcome would be that I would no longer suffer from depression or that my depression would improve’ (P27, f, 26y).

‘Being able to do normal things, like going to work, for example. That would be really useful’ (P13, f, 29y).

Participants expected improvements as a result of laparoscopy, such as **a feeling of agency over one’s body**, **increased quality of life**, and **courage to face life**.

‘Well, I really hope […], it sounds stupid like that, to feel more free in my own body’ (P09, f, 28y).

‘I really hope that my quality of life will be much, much, much better’ (P06, f, 25y)

‘Wanting to live again and to keep on living. I’m really at a point now where I say that I don’t want to live like this anymore. Because it’s becoming unbearable’ (P27, f, 26y).

Another prevalent expectation was to be able to **pursue daily and leisure activities independent of menstrual period**. Engaging in spontaneous and pleasure-oriented activities was anticipated to enhance quality of life.

‘Simply being able to make plans, exercise, meet friends, regardless of my period’ (P27, f, 31y).

Participants also expected **reduced need for and improved efficacy of pain medication** for persistent endometriosis-related complaints.

‘And I hope [pain medication use] will decrease significantly or at least that the pain medication will help then’ (P15, f, 28y).

#### Treatment

Several participants expected **to receive a diagnosis** and **a post-operative treatment schedule**. Some participants also expected this to lead to **self-compassion **and being kinder to themselves.

‘For one thing, the certainty of knowing where this is coming from, what I’m suffering from. Simply being able to put a name to the condition is an important factor’ (P25, f, 33y).

‘Once it’s confirmed, I’ll suddenly have the golden thread to guide me further along’ (P10, f, 42y).

‘Being able to be more considerate towards myself because I’ll know the reason, that it’s not psychosomatic’ (P10, f, 42y).

Participants also expected **removal of endometrial tissue** and **no side effects **from laparoscopy.

‘Them finding something that can be removed as much as possible. Or preferably completely’ (P02, f, 33y).

‘Not having any major side effects, afterwards’ (P31, f, 35y).

Some participants just expressed **curiosity **regarding treatment but no direct expectations.

I’m very curious about the results because doctors always tell me that everything is alright physically.

Other participants explicitly expressed **neutral expectations** regarding laparoscopy and their post-operative quality of life, wishing to avoid potential disappointment while still hoping for improvement.

‘Well, I’m not the type to have great expectations. Because otherwise I’ll just be disappointed if nothing changes. That’s why I prefer to think, well, let’s just see and hope that it will go well, of course’ (P22, f, 33y).

### Negative expectations

#### Complaints and disability

Some participants expected **complaints to persist **after laparoscopy. One participant mentioned expecting a post-operative** increase in complaints**.

‘To be honest, I don’t think it will help much’ (P26, f, 29y).

‘Or the pain becoming even worse, maybe’ (P27, f, 26y).

Based on previous treatment experiences, some participants expected their **complaints to be invalidated** by healthcare providers.

‘Not being taken seriously afterwards’ (P07, f, 39y).

#### Treatment

Regarding laparoscopy, some participants expected **side effects** (e.g., CO^2^-related shoulder pain, pain at the penetration point) and **scarring**. Because of anticipated side effects, some participants also expected **prolonged recovery from post-operative pain**.

‘Yes, well, some things that will happen after the laparoscopy. Pain during urination. Pain in the abdominal area’ (P03, f, 36y).

‘My body also taking a long time to completely […] get back to normal and recover as I’d like it to’ (P17, f, 31y).

Based on a positive experience with inpatient laparoscopy, one participant expected an **outpatient laparoscopy to be inappropriate** and feared a lack of medical attention and support.

Some participants also expected **not to receive a diagnosis** and an associated **post-operative treatment schedule**.

‘That endometriosis isn’t confirmed to the extent it’s currently predicted, and I’m back to square one, still searching for a guiding thread’ (P07, f, 36y).

Several participants mentioned **anxieties** but no explicit negative expectations regarding laparoscopy. Some women feared **surgical complications** such as intra-operative organ damage.

‘And I haven’t slept in days because I’m just very anxious about this operation as such’ (P35, f, 36y).

‘And that’s also a very big fear, […], that I might be somehow damaged because something happens during the operation’ (P17, f, 31y).

### Facilitators and barriers

Facilitators and barriers are divided into *short-term* and *long-term quality of life*.

### Facilitators

When asked about facilitators for post-operative quality of life, participants mentioned positive expectations coming true as facilitators, specifically *“*receiving a diagnosis,” “validation of complaints”, " improvement of complaints”, “improved mental health”, “absence of complaints”, “bodily agency”, “the ability to pursue daily and leisure activities independent of menstrual period”, “becoming pregnant”, “removal of endometrial tissue” and a “post-operative treatment schedule*”.* Further facilitators are listed below.

#### Short-term quality of life

Participants mentioned that **rapid recovery from post-operative pain **would be necessary for their post-operative quality of life.

‘Making good progress after the operation. Like, it not taking long until I’m not in pain anymore’ (P29, f, 34y).

Some participants felt inadequately informed about laparoscopic treatment and would have liked **more treatment and patient information** (e.g. about side effects, post-operative pain management, and surgical results). Furthermore, participants wished for more **professional support and medical aftercare**, especially from gynaecologists in private practice. Having a qualified contact person to provide reassurance in an emergency was anticipated to be helpful for post-operative quality of life.

‘When you feel well-informed and […], basically, they just explain what has happened and tell you that, okay, these are normal complaints that may occur afterwards’ (P11, f, 31y).

‘Also, my gynaecologist’s further support and supervision is very important, I think […]. Being, like, taken care of a bit and not left alone’ (P12, f, 28y).

Some participants expressed dissatisfaction with current treatment options and wished for **new complementary treatments** alongside existing ones. Referring to past treatment experiences, one participant also mentioned that an **inpatient stay** would be more suitable to facilitate post-operative quality of life.

‘They usually tell you that, afterwards, you’ll either have to get pregnant or take the pill. And I’m not really keen on the pill because I’ve always had adverse reactions. And it would be great if […] it turns out, through research or whatever, that there is a third alternative’ (P12, f, 28y).

Participants anticipated that **rest** and **light exercise** would positively impact their post-operative quality of life.

‘Rest, definitely, and giving yourself time’ (P15, f, 28y).

‘Trying to keep as active as possible, within reason, of course’ (P17, f, 31y).

Some participants mentioned that **treatment adherence** would have a positive impact.

‘Well, complying with what the doctors say or with the action plan, doing what is necessary’ (P19, f, 40y).

#### Long-term quality of life

Participants expressed that **awarenessandunderstanding of the treatment procedure** would contribute to better post-operative quality of life. Particularly, some participants referred to good experiences with their clinic or having knowledge of the treatment process from their own professional experience.

'So, I’m familiar with the process through my work, and that’s why I don’t really have any fears about it. Or any negative attitudes.'

Participants stated that **supportive nutrition/diet** (e.g. anti-inflammatory) and **goodtolerability of supplementarty hormon treatments** would positively impact their post-operative quality of life.olerability of supplementary hormone treatments

‘Positive aspects are, above all, adjusting my diet to my endometriosis’ (P18, f, 24y).

**Social support** was also cited as positively impacting post-operative quality of life, including a supportive work environment, peer-group exchanges, and understanding and supportive family and friends.

Many participants reported frustrating experiences with their gynaecologists, which included invalidation of their complaints and disregarding complaints related to endometriosis over the years. Participants wished for **more gynaecologists specializing in endometriosis** and **greater awareness for endometriosis** to prevent years of waiting for a correct diagnosis and experiences of invalidation of endometriosis-related complaints and disability.

‘Only that more gynaecologists should be aware of this issue’ (P29, f, 34y)

‘I just think this study is super important to make people aware. I also talked about it with friends and told them, hey, I’m having an operation because I might have endometriosis and then they asked what that is and I think a woman, well, every woman should know what it is and gynaecologists should provide more information on this issue’ (P21, f, 34y).

In addition, some participants anticipated that **collaboration among healthcare providers** would be a positive impact factor, mentioning how a ‘manager’ for pre- and post-operative treatment could facilitate coordination and communication between healthcare providers, providing more satisfactory patient care and support.

‘That’s what I feel is missing now in this laparoscopy process: a coordination role for the future as well, what happens next, who can support me, also to inform doctors because they don’t have any information at all, they don’t know anything’ (P10, f, 42y).

### Barriers

#### Short-term quality of life

Participants anticipated **post-operative pain** and **scarring** to present barriers to post-operative quality of life.

Some participants indicated that **feeling poorly cared for during treatment** could negatively impact their post-operative quality of life.

‘It’s mostly about the treatment as such at the hospital […]. If you’re not being cared for properly, I think this will have some kind of psychological effect’ (P13, f, 29y).

Some participants perceived a potential **lack of patient information** and **subsequent treatment information** and **lack of treatment adherence** as a barrier to a positive outcome.

‘The gynaecologist, for example, doesn’t really give you much information, it’s not very much, basically. But all of these details that are really important, that is, what can happen and what it will be like and so on, is something I found out by doing my own research’ (P17, f, 31y).

‘Not listening to doctors’ advice’ (P29, f, 34y).

Further barriers to post-operative quality of life were **insufficient rest** after surgery.

**Negative expectations** were also cited as barriers to post-operative quality of life. Particularly participants with prior **negative treatment experiences** mentioned worries about post-operative pain relief or a potential increase of complaints.

‘Maybe still being in pain because it’s in your head. Just because of anxiety that builds up over time. If that’s a factor somehow, sort of like the placebo effect’ (P17, f, 31y).

‘Well, I’ve had an operation before […] and I felt worse afterwards, the pain became even more severe’ (P27, f, 26y).

####  Long-term quality of life

Several participants anticipated that **not receiving a diagnosis** could negatively impact their post-operative quality of life. In this case, participants feared experiencing invalidation and needing further treatments. Some further stated that receiving an **unexpected diagnosis** would negatively impact their post-operative quality of life because of its potentially scary character.

‘The worst thing, which would really affect me, would be if they didn’t find anything’ (P30, f, 31y).

‘Well, my main concern is that there’s something physical that makes it even worse’ (P22, f, 30y).

One participant said being labelled **“a complex treatment case”** would negatively impact their post-operative quality of life, as it would be associated with unsatisfactory improvements in complaints and disability and further necessary treatments.

‘If they tell me, “Ms [last name], yours is not a simple case”’ (P07, f, 39y).

Other participants cited **incomplete endometrial tissue removal**, **persistent complaints**, and an **inability to get pregnant** as negative impact factors.

‘Well, you see mothers with their children every day and this would be even worse for me’ (P27, f, 26y).

**Mental health issues** (e.g. experiencing feelings of depression and emotional instability), **stress**, an **unhealthy lifestyle**, and **lack of social support** were also identified as barriers.

‘Depending on what results I’ll get after the operation. Just slipping deeper into this depression’ (P27, f, 26y).

‘If I didn’t have the support of my husband and family, who will just have to help me after the operation until I’ve recovered’ (P15, f, 28y).

## Discussion

### Summary

We aimed to understand patient expectations regarding laparoscopy and post-operative quality of life and to identify factors that act as facilitators of or barriers to post-operative quality of life. Three top-level categories – *complaints*, *disability*, and *treatment* – were deductively assigned for positive and negative expectations. Following this, eighteen subcodes were identified for positive expectations and ten for negative expectations, inductively. Two top-level categories, short and long-term quality of life, were inductively identified, with 16 subcodes for facilitators and 15 for barriers. Positive expectations included significant improvement or absence of complaints, receiving a diagnosis, and the ability to pursue daily and leisure activities independent of menstrual period. Participants also named negative expectations, such as persistent complaints, feeling invalidated, and side effects. Some positive and negative expectations regarding laparoscopy and post-operative quality of life were also identified as facilitators of or barriers to post-operative quality of life. Concerning further facilitators and barriers, two main themes became apparent: (1) factors influencing *short-term quality of life*, including lack of rest and insufficient treatment and patient information; (2) factors influencing *long-term quality of life*, including more gynaecologists specializing in endometriosis, and heightened public awareness.

### Comparison with existing literature and clinical implications

Frequently reported complaints such as pelvic pain, dysmenorrhoea, dyspareunia, dysuria, and dyschezia align with previous quantitative and qualitative findings [6, 30] and are recognized as the main symptoms of endometriosis. Symptoms not or less often discussed in the literature include accompanying complaints such as gastrointestinal symptoms, water retention, and cystitis. Areas being affected by endometriosis-related disability, such as mental health, ability to work and sexual intimacy, are also consistent with previous studies [9, 31, 32]. Our findings once again underscore the physical and psychological burden on women with endometriosis, highlighting the imperative for interdisciplinary treatment approaches beyond medical interventions such as surgery.

No other qualitative studies have previously examined expectations towards laparoscopy in people with endometriosis. Patients mentioned concurrent positive and negative expectations towards laparoscopy and post-operative quality of life, which emphasizes the multidimensional concept of expectations [33], which also aligned with the baseline results of our ROXWELL study (Melrose et al., 2024). Identified positive expectations suggest that patients place much hope in the surgery, anticipating substantial improvements in complaints or disability following laparoscopy. Subsequently, they expect enhanced quality of life, such as the ability to engage in daily and leisure activities independently of their menstrual period. Another prevalent positive expectation was to receive a diagnosis and a post-operative treatment schedule, likely because many participants had previously experienced diagnostic delays and years of uncertainty. A comprehensive review by Young et al. [10] underscores the significance of validating patient complaints, especially after they have previously faced dismissal or invalidation from their social environment or healthcare professionals. Providing a label for the condition, initiating an appropriate treatment schedule, and guidance for future treatment opportunities can significantly enhance the patient’s overall well-being. Some participants positively anticipated a complete absence of complaints. However, given that 20 to 30% of patients report significant enduring post-operative complaints, and considering the established chronic nature of the disease [15, 34, 35], such an expectation appears unrealistic. Positive expectations corresponding to the placebo effect can enhance treatment outcomes for various medical conditions [36], but they may become problematic if they are unrealistically positive. Literature suggests that unrealistic pre-operative expectations that do not align with medically realistic treatment outcomes can result in disappointment and dissatisfaction [37, 38] and feelings of depression [39], subsequently contributing to a more negative perception of one’s complaints [40]. Providing more precise information on realistic treatment outcomes while highlighting the benefits of laparoscopy could help manage unrealistically positive expectations and mitigate post-operative disappointment with treatment outcomes.

Some participants negatively expected persistent complaints, post-operative side effects, such as CO2-related shoulder pain, and prolonged recovery from surgical pain. Previous quantitative studies have shown that such negative expectations about potential side effects are associated with poor treatment outcomes due to nocebo effects [41, 42]. Providing information about the nocebo effect reduces negative expectations and positively impacts treatment outcomes [43, 44]. Therefore, endometriosis patients with negative expectations for laparoscopy may especially benefit from pre-operative information about the nocebo effect to minimize its risks and, in turn, improve treatment outcomes.

Positive and negative expectations were sometimes verbalized as hopes and fears, respectively. It seems that patients distinguish between expectations and hopes along the lines of: “What can I expect and what would I hope for?“. Conceptually, hopes and expectations are separate constructs [45]. However, it is challenging to grasp because of its known relation. Previous research has linked hope to the concept of expectations, understanding hope as an emotional component of expectations [46]. Anxieties are also integral to the concept of expectations in the sense of negative future-oriented cognitions regarding treatment outcomes [47]. The coexistence of hopes and fears within the framework of expectations emphasizes the complexity of patient expectations. Hopes and realistic expectations should be explicitly differentiated in future studies. During participant checking, one participant stated that they wanted to expect improvement, but that endometriosis had often brought her back to reality in the past, thus making positive expectations unrealistic for them. This shows that cognitions fluctuate between hope and scepticism. Health professionals should be advised to take patient anxieties and fears seriously, be empathetic, and explore the nature of the fears involved. Proactively preventing anxieties and fears could also be achieved through effective/enhanced treatment and comprehensive patient information. Patient and treatment information could also prevent the risk of misinformation (e.g., through online self-research) and exploit the potential of placebo effects through good medical communication. As reported by Lukas et al. [48] and consistent with our identified facilitating factor, participants anticipated that enhanced treatment information, including guidance on managing post-operative symptoms, would positively impact their post-operative quality of life, particularly in the short term.

Concerning further facilitators, women often wished for more gynaecologists who specialized in endometriosis to avoid diagnostic delays and experiences of invalidation. In their comprehensive review, Young et al. [31] highlight women’s experiences with health professionals with limited or no knowledge of endometriosis and conclude that improving education and knowledge about endometriosis would reduce diagnostic delay and enhance women’s care experience. Additionally, in our study, participants expressed that greater public awareness of endometriosis could indirectly enhance their quality of life as it would likely increase sympathy and understanding in their social environment, including from employers. Further facilitators included social support, physical activity, and nutrition [49–51]. Newly identified factors affecting short- and long-term post-operative quality of life include quick recovery from post-operative pain, treatment adherence, and collaboration among healthcare providers. Newly identified barriers to quality of life included post-operative pain and scarring, insufficient rest, lack of social support, and personal feelings of stress.

### Strengths and limitations

Our findings provide insights into expectations related to laparoscopy and facilitators of and barriers to post-operative quality of life. Notably, this is one of the first qualitative studies to assess expectations in patients with endometriosis. Structural content analysis followed strict methodological standards for qualitative research. Participant checking increased patient involvement, clinical relevance, and the credibility of findings. Sampling may have been selective because patients with mostly negative expectations towards laparoscopy would not consider surgery, resulting in a more extensive range of positive pre-operative expectations than negative ones. Some interview questions were similar, so the participants’ answers sometimes overlapped. Future interview questions should be phrased more selectively to avoid repeating answers.

## Conclusion

Positive and negative expectations exist concurrently, underscoring the multidimensionality of expectations. Patients emphasized the significance of more comprehensive pre-and post-operative patient and treatment information. Information should encompass (1) enhanced information about the pre- and post-operative treatment process to alleviate treatment-related fears, (2) clear information about realistic treatment outcomes to shift unrealistic expectations towards more realistic ones, and (3) guidance for future treatment opportunities. Providing additional information about nocebo effects may help prevent adverse effects in patients with negative treatment expectations.

Addressing the need for more endometriosis specialists may help mitigate diagnostic delays and experiences of invalidation, while greater public awareness of the condition may contribute to improved quality of life through greater sympathy and understanding. The facilitators and barriers identified in this study show that patients are experts regarding their disease. They are aware of practical ways to manage their complaints and are very clear about what is helpful or not in terms of their quality of life after laparoscopy. For this reason, patients should be actively involved in clinical research and developing intervention concepts.

## Electronic supplementary material

Below is the link to the electronic supplementary material.

Supplementary Material 1

Supplementary Material 2

Supplementary Material 3

## Data Availability

Due to the sensitive nature of the interview scripts and privacy concerns of study participants, we will share qualitative data only upon reasonable request by the corresponding author.
