# Supplementary material for: Pre-operative expectations in patients with endometriosis – a qualitative interview study
Source: BMC Womens Health. 2025 Apr 28;25:209. doi: 10.1186/s12905-025-03686-3 (PMC12039098; doi:10.1186/s12905-025-03686-3)
Supplement: Supplementary file 1 — Supplementary Material 1 [file 12905_2025_3686_MOESM1_ESM.docx]

| **Positive expectations** | | | | | | |
| --- | --- | --- | --- | --- | --- | --- |
| Theme | Thematic Subcode | Exemplary quotations | | | | |
| **Complaints and disability** | Complaint improvement | *‘Exactly, and the hope that there will be less pain afterwards. So, I don’t really expect that there will be no complaints anymore, but I just hope that they will be at a lower level so that I’ll be less impaired and be in less pain maybe’* (P11, f, 31y*)*  *‘Less pain in everyday life’* (P29, f, 34y*)* | | | | |
|  | Absence of complaints | *‘Hopefully, being able to carry on living as a woman without being in pain’* (P02, f, 33y*)*  *‘The pain being gone. A long-term solution being found’* (P07, f, 39y*)* | | | | |
|  | To get pregnant and become a mother | *‘And hopefully, eventually, an easier time getting pregnant in the future’* (P29, f, 34y*)*  *‘That our desire to have children works out’* (P35, f, 36y) | | | | |
|  | General improvements in disability | *‘Yes, impairments being reduced or eliminated’* (P35, f, 36y) | | | | |
|  |  | *‘Just feeling less impaired’* (P09, f, 28y) | | | | |
|  | Improvement in mental health | *‘I don't want to say it makes me depressed, but it's just burdensome to live constantly with this pain. And I wish that it just goes away’* (P35, f, 36y) | | | | |
|  |  | *‘Yes. I said that I want life to go on, that I’m thinking positively, and that I don’t spend all the time thinking about what has happened to me. What this thing might be’* (P06, f, 25y) | | | | |
|  | Improvement in physical disability | *‘I expect that afterwards, I’ll once again be able to use the toilet without any problems and move around during everyday activities’* (P18, f, 24y) | | | | |
|  |  | *Well, no longer being confined to bed when my period starts* (P13, f, 29y) | | | | |
|  | Ability to work | *‘Well, of course I hope for major improvement, because, like I said, every month I can’t do anything for a few days. I don’t work, and I don’t feel well’* (P12, f, 28y) | | | | |
|  |  | *‘That you can also simply pursue a normal work profession’* (P17, f, 31y) | | | | |
|  | Improvement in  sexual intimacy | *‘Also, for example, being able to get more enjoyment out of sexual intercourse again’* (P31, f, 35y)  *‘Well, really, sexual intercourse improving again’* (P03, f, 36y) | | |  |  |
|  | Reduced strain on romantic relationships | *Not suddenly becoming incapacitated all the time and being able to sleep with my partner again.* (P07, f, 39y)  *‘I hope that my sex life will be better again. (…) our relationship really suffers (…) when I’m always in pain’* (P09, f, 28y) | | | | |
|  | A feeling of agency over one’s body | *‘I’m being completely serious here, because I believe I’ll be more free and better able to do many things again’* (P10, f, 42y*)*  *‘I just hope that this will make me safer again, fitter and more able to stand on my own two feet’* (P17, f, 31y) | | | | |
|  | Increased quality of life | *‘I expect an improvement of quality of life for any outcome of the operation, simply because of this validation of my pain’* (P18, f, 24y)  *‘When I have my period, the days shortly before and after, my quality of life will be better in any case because I won’t be in pain all the time’* (P21, f, 24y) | | | | |
|  | Courage to face life | *‘Having more desires and objectives again, other desires and objectives. That no one can say that I only wish to live without pain, but also that I can see other qualities again’* (P27, f, 26y)  *‘And I just hope that I feel like living again and can enjoy my everyday life a bit more’* (P06, f, 25y) | | | | |
|  | Pursure daily and leisurely activities independent of menstrual period | *‘Being able to manage my everyday life normally. Right now, it’s like this: When I’ve arranged to meet friends in the evening, and I know that I might get my period that day, I don’t know if I can confirm that I’m coming because I just won’t feel well then. Because I’ll be in pain’* (P13, f, 29y)  *‘Not always having to arrange my whole life around it’* (P17, f, 31y) | | | | |
|  | Reduced need for pain medication | *‘Maybe having to take less pain medication’* (P35, f, 36y)  *‘You take a lot of pain medication, and it's just not nice. It just becomes so that you can say okay, a hot water bottle is enough now’* (P17, f, 31y) | | | | |
| **Treatment** | To receive a diagnosis | *‘But also, insight. Like, knowing better how I am currently doing. And if this pain really is connected to this’* (P03, f, 36y)  *‘This is what I think about the most, that I want to have certainty, what exactly, how bad it is, because at the moment there are many different diagnoses and estimations but there’s nothing among those that’s really certain’* (P17, f, 31y) | | | | |
|  | To receive a postoperative treatment schedule | *‘Having a solution of how to go on living with it. The pain being gone. A long-term solution being found’* (P07, f, 39y)  *‘Having a plan afterwards of how to deal, well, I mean, it can’t be cured but that I’ll know at least, okay, what’s the best I can do about it now’* (P17, f, 31y) | | | | |
|  | Self-compassion | *‘And just the peace of mind because I know it’s really there, and there isn’t anything else in my body that always hurts but that I ignore because I think it might be endometriosis’* (P18, f, 34y) | | | | |
|  | Removal of endometrium tissue | *‘I hope that the cysts can be removed’* (P14, f, 30y)  *‘And if they find something, they can cut it away’* (P13, f, 29y) | | | | |
|  | No side effects | *‘To be honest, I’m not afraid that I’ll suffer any adverse reactions or long-term effects’* (P21, f, 24y) | | | | |
|  | Curiosity | *‘I've been wondering, (…), whether the other symptoms I have because of premenstrual syndrome, whether they will also change. And I'm a bit curious about that because I've read different things about it’* (P23, f, 28y)  *‘I'm really looking forward to the result because the doctors always say that organically everything is fine’* (P29, f, 34y) | | | | |
|  | Neutral expectations | *‘I don't really expect much, to be honest. Of course, I hope it gets better. But the expectation itself is kind of neutral, more like, let's see’* (P22, f, 30y)  *‘Well, I don't really have any negative expectations of the procedure itself. This is my second one, and this time, it will be performed on an outpatient basis’* (P29, f, 34y) | | | | |
| **Negative Expectations** | | | | | | |
| Theme | Thematic codes | | Exemplary quotations |  |  |  |
| **Complaints and disability** | Persistence of complaints | *‘That, in the end, nothing will happen, and it will still hurt so much’* (P23, f, 28y)  *‘That this could actually bring no improvement’* (P17, f, 31y) | | | | |
|  | Increase in complaints | *‘The negative expectations would be that (…) I would have even more pain than before’* (P27, f, 26y) | | | | |
|  | Invalidation of complaints | *‘That, once again, nothing will be found. And I’ll continue to be in pain, and sometimes they don’t ‘believe me when I say I’m in pain’* (P26, f, 29y) | | | | |
| Theme | Thematic code | Exemplary quotations | | | | |
| **Treatment** | Side effects | *‘Well, my negative thoughts are especially that I definitely won’t be well immediately after the operation, and I am relatively apprehensive about what complications there might theoretically be afterwards’* (P18, f, 24y)  *‘Fear that you might have a lot of pain after the operation because of the air and so on’* (P12, f, 28y) | | | | |
|  | Prolonged relief from surgical pain | *‘I’ve heard that I’ll be put on sick leave for eight days. Being bedridden for those eight days. I really hope that won’t happen’* (P13, f, 29y)  *‘That it takes quite a long time to heal’* (P08, f, 34y) | | | | |
|  | Outpatient laparoscopy to be unsuitable | *At this clinic, where I’ll have the operation, it’s an outpatient procedure. When I heard that for the first time, I could hardly believe it. Because this operation is not very, it’s not a minor one. Well, I think this makes me a bit sceptical, and I also don’t know yet how I’ll get home right after the operation, it’s on the second floor, and there’s no lift. These are just, like / Yes, these are my personal feelings, that such an operation really isn’t suitable for being an outpatient procedure’* (P28, f, 24y) | | | | |
|  | Not to receive a diagnosis | *‘They’ll carry out the operation, and then afterwards I’ll wake up, and they’ll tell me “Unfortunately, we didn’t find anything’* (P02, f, 33y)  *‘Afraid that nothing will be found. So, no endometriosis’* (P09, f, 28y) | | | | |
|  | Postoperative treatment schedule | *‘Endometriosis not being confirmed at such a level as is predicted now, and I’ll have to start at zero again and keep looking for causes**’* (P10, f, 42y) | | | | |
|  | Anxieties | *‘I'm a bit scared of it (…). I don't know what kind of pain to expect. And not knowing where all the tissues are located scares me too’* (P35, f, 36y)  *‘So, I'm definitely scared’* (P17, f, 31y) | | | | |
| **Facilitators** | | | | | |  |
| Theme | Thematic code | Exemplary quotations | | | |  |
| **Short-term quality of life** | Rapid recovery of operative pain | *‘Everything healing well. Being in less pain afterwards. Recovering relatively quickly’* (P13, f, 29y)  *‘And that, of course, I take it all in well and that everything actually heals well as it should’* (P33, f, 24y) | | | |  |
|  | Patient and treatment information | *‘To get information material (…). I think that would be very good’* (P25, f, 33y)  *‘I would like to have another conversation before I go under the knife’* (P24, f, 43y) | | | |  |
|  | Professional support and medical aftercare | *‘And, of course, the follow-up care must be appropriate and good* (P28, f, 24y)  *‘Well, it would be important to me, even just the consultation with the surgeon afterwards. Like, it wouldn’t be helpful if I didn’t know exactly when I’d be allowed to do things again, like start exercising, or how long I’d not be allowed to lift things, especially regarding my job’* (P15, f, 18y) | | | |  |
|  | New complementary treatments | *‘Maybe also getting scar treatment so the scars heal well and stuff like that’* (P03, f, 36y)  *‘But I think a lot more is possible; opportunities must be researched and must also be wanted to be wanted’* (P29, f, 34y) | | | |  |
|  | Inpatient stay | *‘I would somehow wish that this would perhaps be an inpatient procedure where you really have a thorough look’* (P22, f, 30y) | | | | |
|  | Rest | *‘Being able to really relax at home and get better’* (P35, f, 36y)  *‘Simply being mindful of your body but also your mind. Just relaxing and taking on this process in as good a state of health and mind as possible’* (P31, f, 35y) | | | | |
|  | Light exercises | *‘Maybe also being able to slowly and carefully, after consultation with my doctor, start exercising again’* (P15, f, 28y)  *‘Well and to some extent movement'* (P29, f, 34y) | | | | |
| Theme | Thematic subcode | Exemplary quotations | | | | |
| **Long-term quality of life** (Facilitators) | Positive expectations | *‘To think positively that it will be good. (...) Imagine what it will be like afterwards without this pain. I just try to think positively, to say what doesn't belong there, that just must come out. And I'll definitely feel better afterwards‘* (P25, f, 33y)  *‘I also don’t think there will be any severe pain afterwards‘* (P30, f, 31y) | | | | |
|  | Supporting nutrition/diet | *‘Trying to eat... let’s call it anti-inflammatory food. There is food that contains natural anti-inflammatory substances* (P21, f, 24y)  *‘I have actually decided to pay close attention to my diet (…). And I'm trying to switch to a light diet after the operation’* (P02, f, 33y) | | | | |
|  | Tolerating supplementary hormonal treatments | *‘Tolerating this hormonal IUD well, and the whole thing not being as bad as in the last few years’* (P08, f, 34y) | | | | |
|  | Social support | *‘Definitely that I’m being supported by all my friends who’ve already said (laughs) they would come by and do everything I need when I’m unable to move or something’* (P17, f, 31y)  *‘I would say that you’re in an environment where you feel safe and like you’re being helped. This means being with family or really with, well, with relatives’* (P18, f, 24y) | | | | |
|  | More gynaecologists specializing in endometriosis | *‘I have suspected that I have endometriosis, for years now, and I always felt belittled a bit by my former gynaecologists’* (P13, f, 29y)  *‘I had my first period when I was ten years old. I've always had difficulties since then, and I've always felt bad. And not a single gynaecologist ever had the idea to say, man, maybe this should be checked out’* (P19, f, 40y) | | | | |
|  | Greater awareness of endometriosis | *‘And yes, that there are more endometriosis centres and a better choice of appointments and yes, simply that the issue becomes more widely known. That’s what I would wish for’* (P29, f, 28y)  *‘I think it's just this general awareness of endometriosis’* (P12, f, 28y) | | | | |
|  | Collaboration among healthcare providers | *‘I just talked to my gynaecologist, she was... when I said that I need an appointment for follow-up care after the laparoscopy, her personnel were somehow overwhelmed and didn’t know how to proceed with giving me an appointment for that. This means that, overall, there needs to be another type of link between doctors or a, let’s call them manager, who can bring everything together’* (P10, f, 42y) | | | | |
| **Barriers** | | | | | | |
| Theme | Thematic subcode | Exemplary quotations | | | | |
| **Short-term quality of life** | Postoperative pain | *‘When the pain doesn’t decrease or when it doesn’t heal properly after the operation’* (P13, f, 29y)  *‘Only, I don’t really want the pain afterwards. I know someone who also has this, endometriosis, she told me she was in pain for four days after the laparoscopy and had to lay in bed, and I don’t want that, but it’s necessary’* (P21, f, 24y) | | | | |
|  | Scarring | *‘I think if scar tissue develops or if there is severe scarring. And you have problems with the scars or healing. (...) I think this could really cause serious limitations for me’* (P09, f, 28y)  *‘However, parts of the skin are cut open and also, well, a lamp is put through the belly button, and I think the changes in my body – because there will probably be scars – could impair my quality of life’* (P18, f, 24y) | | | | |
|  | Feeling poorly cared for during treatment | *‘It’s mostly about the treatment as such at the hospital [...]. If you’re not being cared for properly, I think this will have some kind of psychological effect’* (P13, f, 29y) | | | | |
|  | Missing or incomplete treatment and patient information | *‘During my preoperative talk with my gynaecologist, I was only informed of my upcoming procedure bit by bit, which made it difficult for me to understand the entire process’* (P13, f, 29y)  *‘The operation is now performed outpatient, and they always say no; it must be done on an inpatient basis (…). And it's all like that, there's so much unclear information’* (P17, f, 31y) | | | | |
|  | Inadequate pain-related rest behaviour | *‘Well, the employer may be telling me that I’d have to be back at work by such-and-such time. Like, this would really be something to put some pressure on me’* (P15, f, 28y)  *‘If there were a New Year's Eve party here (…), it wouldn't affect it well either. So if I had no peace and quiet here. I wouldn't be able to recover sufficiently’* (P15, f, 28y) | | | | |
|  | Negative expectations | *‘But I don't see the reason why I should have less pain if they only do a laparoscopy. (…) I don't expect to feel better immediately afterwards’* (P06, f, 25y)  *‘I've had an operation before. (…). And afterwards, I was even worse, the pain got even worse’* (P27, f, 26y) | | | | |
| **Long-term quality of life** | Not receiving a diagnosis | *‘And the pain still remains because they didn’t find a solution’* (P33, f, 24y)  *‘Well, either nothing can be found, that would, of course, be a disaster’* (P13, f, 29y) | | | | |
|  | Unexpected diagnosis | *‘Yes, I think, just the fear of waking up and maybe being told which body parts are affected and not being aware of that yet. This is... this really is a no-go’* (P31, f, 35y) | | | | |
|  | "A complex treatment case" | *‘Or if it’s progressed so much that they tell me they’re unable to do much. Or if the infertility is so bad that it’s inoperable’* (P35, f, 36y) | | | | |
|  | Incomplete endometrium tissue removal | *‘If the operation isn’t successful. If they maybe can’t reach the inflammation foci to remove them. Or if they’re so deep in the gastrointestinal tract that it’s rather difficult to remove them’* (P35, f, 36y)  *‘If they don’t actually remove anything, I expect basically nothing to change’* (P18, f, 24y) | | | | |
|  | Persistent complaints | *‘When the pain doesn’t decrease’* (P13, f, 29y)  *‘And the pain still remains because they didn’t find a solution’* (P33, f, 24y) | | | | |
|  | Mental health issues | *‘I also know that such psychological factors always play a large role for me. So, if I realise that there’s no progress or if the complaints or the pain persist for a longer time after the operation, this will have an impact on my mental well-being’* (P02, f, 33y)  *‘Well, and now, of course, the psychological burden before operation’* (P28, f, 24y) | | | | |
|  | Unhealthy lifestyle | *‘If I drink alcohol during recovery. Smoking is also something that doesn’t speed up healing but rather slows it down’* (P21, f, 24y)  *‘I'm paying a lot of attention to my diet and doing my exercise, and if I'm getting a bit more careless, that's making things a bit worse’* (P18, f, 22y) | | | | |
|  | Lack of social support | *‘Like, it takes some time until a therapy shows results. If you had no support, this would, of course, not be... not be optimal if you couldn’t (unintelligible), like, couldn’t take it easy and rest enough, yes’* (P15, f, 28y) | | | | |
