# Supplementary material for: Pre-operative expectations in patients with endometriosis – a qualitative interview study
Source: BMC Womens Health. 2025 Apr 28;25:209. doi: 10.1186/s12905-025-03686-3 (PMC12039098; doi:10.1186/s12905-025-03686-3)
Supplement: Supplementary file 2 — Supplementary Material 2 [file 12905_2025_3686_MOESM2_ESM.pdf]

ROXWELL Mixed Methods Cohort Study

Qualitative Interview – Part I

Expectations and Influencing Factors

To do before starting the call:

- Open the *"appointment list for qualitative interviews"* and find the patient's telephone number
- *Find out at what time the next interview should take place → about 2.5 months after the laparoscopy (early/mid/late MONTH)*
- Check if declaration of consent to study participation has been signed

General notes for the interviewer

- Always ask questions in the same order
- If the patient has problems understanding something, the interviewer may repeat the question and, if necessary, change the wording (**Important:** document this and change the wording only slightly)
- If the interviewer has problems understanding something, they may paraphrase what the patient has said (but only in this case)
- Have the patient focus on the subject of the question if there is no answer or if the patient repeats something that has already been said
- Avoid follow-up questions (e.g. if a patient cannot think of something, do not give examples but move on to the next question)
- Reaction to what has been said:
  - Do not provide content-related feedback to patients' answers, do not give hints on coping, processing, etc.
  - Active listening (reactions like "okay", "yes", "hm", "I see") is preferable
  - Saying "thank you for your frankness" is permitted.
  - If patients give only one example, ask again: Is there anything else? Can you think of anything else? (or similar questions, depending on the situation)
- Do not by any means mention the patient's name while audio is being recorded

Qualitative questions on expectations and influencing factors | *Part I*

Hello Ms. XY,
my name is ______ and I'm part of the study team of the ROXWELL study. I'm calling you today for your first interview. This will take about 15 minutes. The following questions refer to your state of health, your expectations regarding the medical intervention (laparoscopy) and to factors that (may) have a positive or negative influence on postoperative development. Since this part of the interview will be recorded and later transcribed word by word, I'm asking you to keep your answers to the following questions as short and to the point as possible. At the end, I will ask you a few general questions about yourself that will not be recorded.

If the appointment is inconvenient for the patient, make a new appointment (in the near future).

Note: new appointment on __________ at __________ → transfer appointment to XXX

Before beginning the interview, I would like you to create a ***new*** *personal code so that we will later be able to attribute the first and second interview to the same person.*

The code will be composed of the following elements (Be careful to use the exact wording. Otherwise, correct any mistakes/clarify any uncertainties):

1. *The* ***last*** *two letters of your father's first name. If you don't know your father's first name, please use your own last name.*
2. *The number of letters of your mothers 's first name. If you don't know your mother's first name, please use your own first name.* → ***Write this down with a leading zero!***
3. *The last two letters of your mother's maiden name. If you don't know your mother's maiden name, please use your own maiden name.*
4. *Your own birthday (only the day without the month and/or year).* → **Write this down with a leading zero!**

CODE: _ _ __ _ _ __

Do you have any further questions right now?

If there are no more questions: *Do you consent to participate in the study and to the associated audio recording? If yes: I'm now starting the recording and I will begin by repeating your* ***code*** *and the* ***question if you consent*** *to take part in the study, which I would like you to answer again.*

START recording

Repeat the code: _ _ __ _ _ __

Do you consent to participate in the study and to the associated audio recording?

At first, we would like to ask you some general questions about your endometriosis-related complaints and any impairment caused by them.

1. Which (endometriosis-related) complaints do you have? *(If only answer is pain, what kind/how does it feel?)*

2. How do your (endometriosis-related) complaints typically develop during your everyday life? *(If answer does not relate to time, then ask: in particular with regard to time, per minute, hour, day, week, month?)*

3. What determines the development of your (endometriosis-related) complaints?

4. Which impairments do you have because of your (endometriosis-related) complaints?

In the following, we would like to know more about your thoughts and expectations regarding the medical intervention (laparoscopy).

5. What is the first thing that comes to your mind when you think about the medical intervention (laparoscopy)?

6. What are the negative expectations you have regarding the medical intervention (laparoscopy)?

7. What are the positive expectations you have regarding the medical intervention (laparoscopy)?

In the following, we will talk about impairments due to your endometriosis-related complaints after the medical intervention.

8. How do you expect your impairments to change after the medical intervention (laparoscopy)?

We are interested in factors that might influence the postoperative development of your impairments both in a positive and negative way.

9. Which factors might have a negative influence on postoperative development?

10. Which factors might have a positive influence on postoperative development?

We would now like to ask you about your quality of life after the medical intervention (laparoscopy).

11. How do you expect your quality of life to change after the medical intervention (laparoscopy)?

12. Which factors might have a negative influence on your quality of life after the medical intervention (laparoscopy)?

13. Which factors might have a positive influence on your quality of life after the medical intervention (laparoscopy)?

14. Is there anything else that you would like to tell us about your experience with the medical intervention (laparoscopy) and your expectations? / ... that is important to you and that you would like to tell us?

Thank you, that was the last question of this part of the interview. I'm now stopping the recording.

Stop the audio recording
