# Supplementary material for: Pre-operative expectations in patients with endometriosis – a qualitative interview study
Source: BMC Womens Health. 2025 Apr 28;25:209. doi: 10.1186/s12905-025-03686-3 (PMC12039098; doi:10.1186/s12905-025-03686-3)
Supplement: Supplementary file 3 — Supplementary Material 3 [file 12905_2025_3686_MOESM3_ESM.docx]

Supplement Table 1. Participant flow chart

- *n* = 2 did not fulfill inclusion criteria (*n* = missing endometriosis diagnosis)
- *n* = 3 were not reached by phone
- *n* = 2 laparoscopy was already performed
- *n* = 1 pregnancy

*n* = 33 final included participants

*n* = 35 participants took part in 1st interview

*n* = 41 interested interview study participants
